# Massa Medicata Fermentata treated spleen deficiency constipation by mediating intestinal microbiota and serum peptide

**DOI:** 10.3389/fcimb.2025.1556915

**Published:** 2025-03-06

**Authors:** Kangxiao Guo, Yuan Tang, Tao Yang, Yongwang Yan

**Affiliations:** ^1^ National Engineering Laboratory for Rice and By-Product Deep Processing, College of Food Science and Engineering, Central South University of Forestry and Technology, Changsha, China; ^2^ Department of Pharmacy, ChangSha Health Vocational College, Changsha, Hunan, China

**Keywords:** Massa Medicata Fermentata, spleen deficiency constipation, intestinal microbiota, SP, VIP, CGRP

## Abstract

**Objectives:**

To investigate the correlation between the treatment of spleen deficiency constipation and the typical brain and intestinal peptides.

**Methods:**

A total of 18 male Kunming mice were randomly divided into three treatment groups (n = 6): normal group (CC), model group (CM), and Massa Medicata Fermentata intervention group (CG). CM and CG were used to establish a spleen deficiency constipation mouse model. After the model was finished, CG was infused with 0.15 g/mL Massa Medicata Fermentata water infusion at a dose of 4 g/(kg·day), twice a day, at 0.4 mL. An equal amount of distilled water was infused in CC and CM for 7 days. The body weight and fecal water content of the mice were monitored during the modeling. Following the intervention, 16S rRNA amplicon sequencing was used to analyze changes in the microflora in the intestinal contents, and serum substance P (SP), vasoactive intestinal peptide (VIP), and calcitonin gene-related peptide (CGRP) levels were determined via ELISA.

**Results:**

The modeling had no significant effect on the weight of the mice, the water content of the mice’s feces was greatly reduced, and the feces were dry and hard. Constipation caused by spleen deficiency can lead to a decrease in serum SP and an increase in VIP and CGRP. After treatment with Massa Medicata Fermentata, SP, VIP, and CGRP all changed. Intestinal microbiota diversity of mice with spleen deficiency constipation, and the dominant microbiota and characteristic microbiota changed, indicating that the intestinal microbiota was unbalanced. After the intervention of Massa Medicata Fermentata, the intestinal microbiota diversity of spleen deficiency constipation mice increased; the dominant microbiota became *Candidatus* Arthromitus, *Lactobacillus*, unclassified Bacilli, *Bacillus*, *Ligilactobacillus*, *Muribaculaceae*, *Bacteroides*, and *Enterorhabdus*; and the characteristic microbiota became *Candidatus* Arthromitus. Through the analysis of characteristic microbiota and serum SP, VIP, and CGRP levels, *Ligilactobacillus* was found to be positively correlated with SP and negatively correlated with VIP, *Akkermansia* and *Streptococcus* were negatively correlated with SP, *Candidatus* Arthromitus was negatively correlated with CGRP, *Akkermansia* and *Candidatus* Arthromitus were negatively correlated with VIP, and *Candidatus* Arthromitus was negatively correlated with CGRP.

**Conclusions:**

Massa Medicata Fermentata can affect the secretion of short-chain fatty acids in the intestine by altering the microecological environment of the intestine, then affect the secretion of serum peptides in mice, and alleviate the spleen deficiency constipation.

## 1 Introduction

Constipation is a prevalent gastrointestinal disorder with a worldwide prevalence of 16%, and in China, 20%–30% of the population is affected according to epidemiological surveys conducted in the United States in 2011 (Chen et al., 2023). The prevalence of constipation is on the rise, with the elderly and female patients being the most affected due to diet, environment, and emotions. Clinical symptoms mainly include infrequent bowel movements (less than three times per week), difficulty in defecating, and dry and hard stools, often accompanied by abdominal pain and distention (Tang, 2017; Xu et al., 2023). Furthermore, it can also result in intestinal dyskinesia and reduced sensitivity, as well as tumors and other conditions that compress the intestinal tract, mechanically impeding peristalsis and reducing smooth muscle tone. Impaired defecatory muscle group activity and weakened peristalsis may also occur, along with intestinal microbiota disorders. Long-term constipation can cause damage to the intestinal wall and lead to the accumulation of toxins in the intestinal tract, resulting in systemic complications.

**Figure 1:**
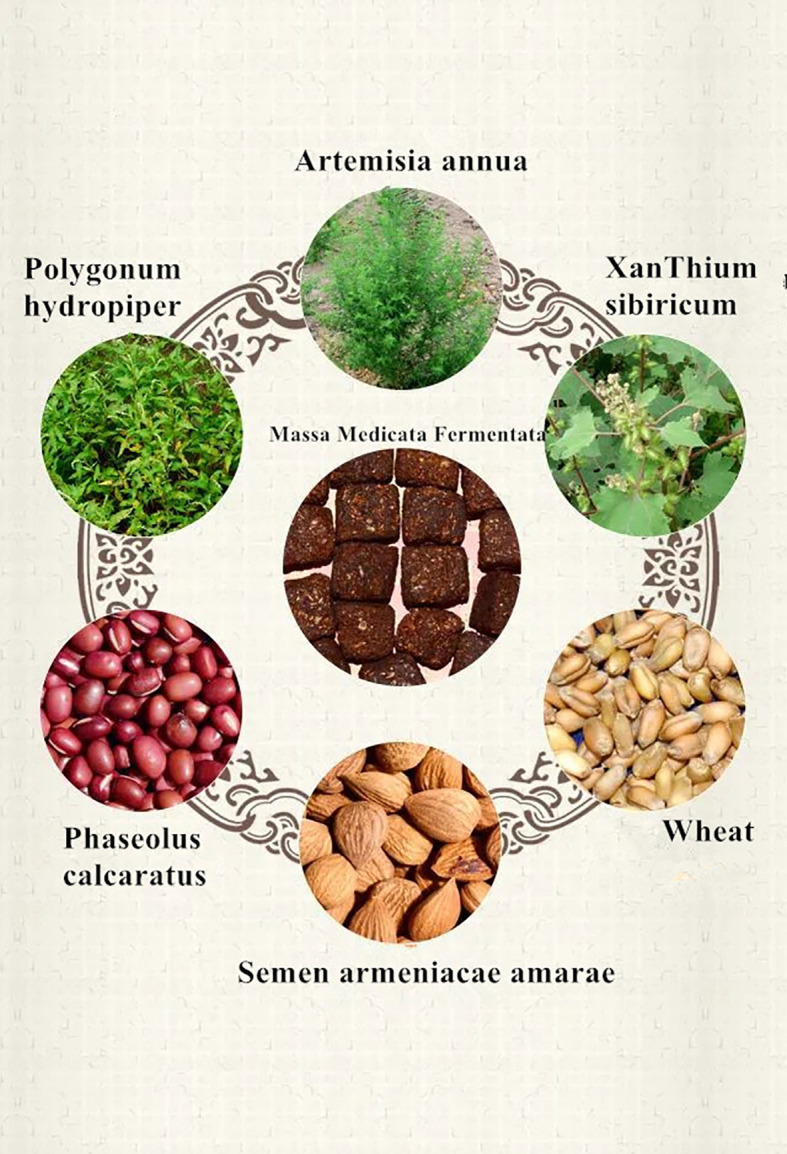
Composition of the Massa Medicata Fermentata.

Currently, Western medicine recommends symptomatic treatment for gastrointestinal diseases. Commonly used treatments include gastric motility drugs and surgery. However, there are limitations to this approach such as single treatment methods and drugs, low safety, side effects, relapse, and many sequelae, resulting in unsatisfactory curative effects. Traditional Chinese medicine (TCM) has significant advantages in treating constipation, with two prominent clinical benefits. Based on the clinical manifestations of constipation, TCM categorizes it as “fullness”, “eructation”, “belching”, and other symptoms. The cause of the disease is often attributed to improper diet, internal injury caused by emotional distress, or spleen deficiency, with liver disorder, spleen dysfunction, and stomach failure as the basic pathogenesis. The disease is in the intestine, which is closely related to the spleen and stomach (Wen and Liu, 2023; Wu and Zhang, 2023). Therefore, the treatment should focus on strengthening the spleen through dietary adjustments. Yi et al. (2023a) found that the Zhishi Daozhi decoction has a good effect on constipation caused by high fat and high protein diet; Massa Medicata Fermentata is mentioned in the “medicinal theory” as a remedy for water retention and stagnation and to strengthen the spleen and warm the stomach. Massa Medicata Fermentata is made from wheat bran as a fermentation medium, with the addition of *Artemisia annua*, spicy *Polygonum*, and *Xanthium* grass (**Figure 1**) . The mixture is then fermented with porridge cooked from crushed bitter almonds and red beans. Massa Medicata Fermentata is one of the most used Qu agents today. Compared with Zhishi Daozhi decoction, Massa Medicata Fermentata not only can treat spleen deficiency constipation but also strengthen the function of the spleen and stomach, treat spleen and stomach weakness, and treat diet stagnation in digestion and regulation. However, the mechanism of Massa Medicata Fermentata in treating spleen deficiency constipation is currently unclear. To investigate the microecological mechanism of Massa Medicata Fermentata in treating spleen deficiency constipation, we based on the theory of the cerebral–intestinal bacteria axis; the project team conducted research on constipation with spleen deficiency mice via reverse transcription polymerase chain reaction (RT-PCR), enzyme-linked immunosorbent assay (ELISA), and other approaches to observe the effects of Massa Medicata Fermentata on intestinal microecology and serum peptide human serum substance P (SP), vasoactive intestinal peptide (VIP), and calcitonin gene-related peptide (CGRP) in mice. This study aimed to provide experimental evidence for the development of Massa Medicata Fermentata as a safe and effective Chinese patent medicine for the treatment of spleen deficiency constipation by examining the influence of SP, VIP, and CGRP levels.

## 2 Materials and methods

**Figure 2:**
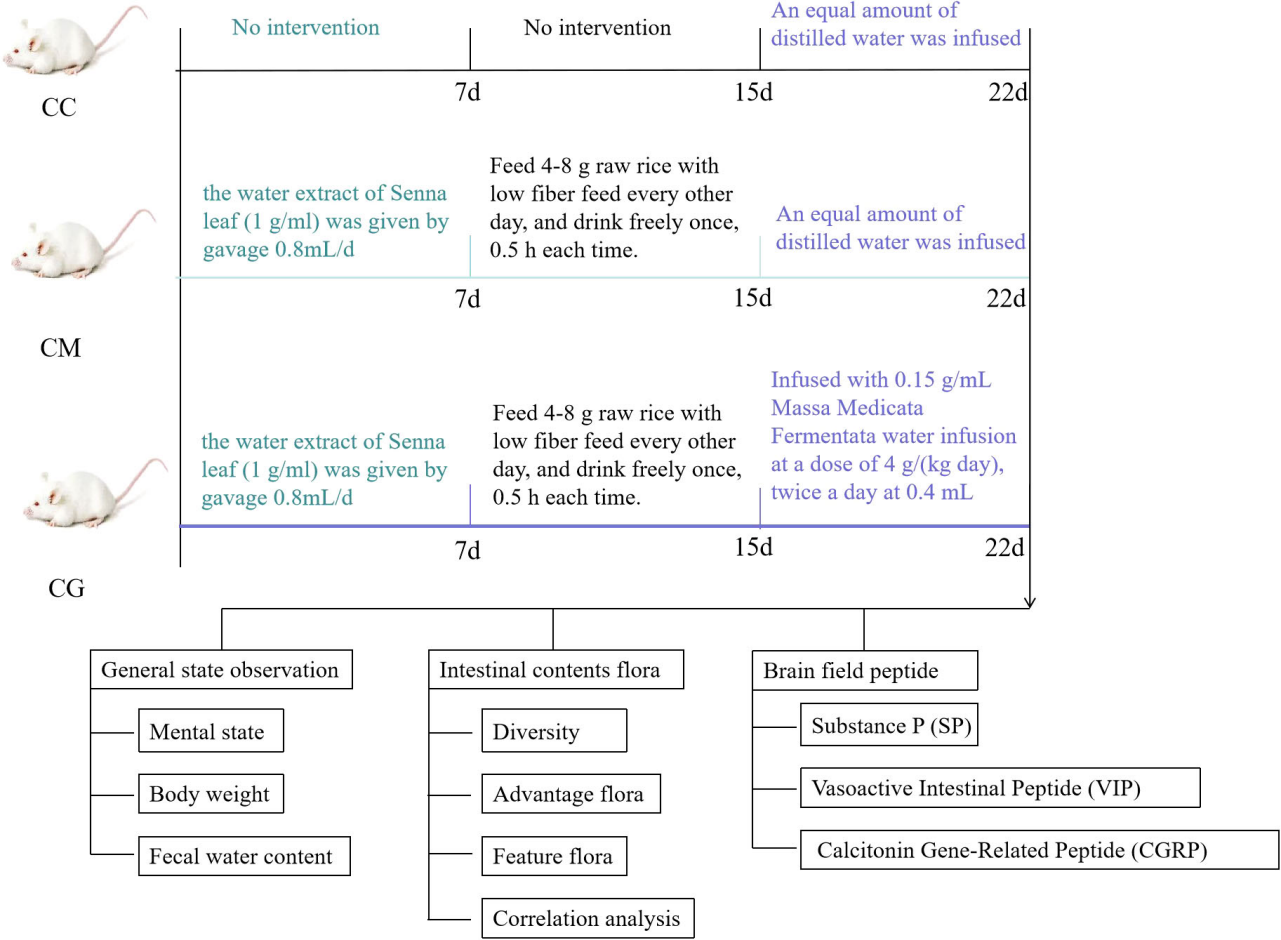
Experimental flowchart.

We studied the effect of Masssa Medicata Fermentata in the adjuvant treatment of spleen deficiency constipation through animal experiments, and the brief experimental flow chart is shown in **Figure 2**.

### 2.1 Animals and procedures

A total of 18 male Kunming mice (4 weeks of age, body weight 20 ± 2 g) were obtained from Hunan Slaccas Jingda Laboratory Animal Company (SYXK(Xiang)2019-0004). All the animal experiments were performed according to the Guidelines of the Laboratory Animal Ethical Committee of Hunan University of Chinese Medicine (LL2022060106). The mice were kept in the environmental control room with temperature maintained at 20°C ± 2°C, humidity of 40%–60%, and a 12-h light and dark cycle in specific pathogen-free conditions. After 1 week of acclimatization, the mice were randomly divided into three treatment groups (n = 6): normal group (CC), model group (CM), and Massa Medicata Fermentata intervention group (CG). The model of spleen deficiency constipation was prepared according to the method of Yi et al (Yi et al., 2023b); from the first to seventh days, mice in the CM and CG groups were given the water extract of Senna leaf (1 g/mL) by gavage at 0.8 mL/day, and on the eighth day, it was withheld to induce hunger and suppress satiety. The abnormal method maintained the state of spleen deficiency. Mice were allowed to feed 4–8 g raw rice with low fiber feed every other day and drink freely once at 0.5 h each time. Based on the spleen deficiency model, the constipation model was established by restricting drinking water and controlling diet, and CC was fed the normal diet. After 15 days of the dietary intervention, the 16-day start CG was infused with 0.15 g/mL Massa Medicata Fermentata water infusion at a dose of 4 g/(kg·day), twice a day, at 0.4 mL. An equal amount of distilled water was infused in CC and CM for 7 days. By the end of the trial, the mice were killed. Their intestines were collected for the follow-up experiments, immediately frozen in liquid nitrogen, and stored at −80°C.

### 2.2 Medicine

The Massa Medicata Fermentata was purchased from the First Affiliated Hospital of the Hunan University of Chinese Medicine. The Massa Medicata Fermentata was poured into a pre-sterilized mortar for full grinding and then prepared into a gastric lavage with a concentration of 0.15 g/mL with distilled water. The liquid was stored in a refrigerator at 4°C for later use.

Reagents used in the determination of intestinal microbial activities were Food and Drug Administration (FDA) approved and purchased from Shanghai Yuanye Biotechnology Co., Ltd. (Shanghai, China). Phosphate buffer, acetone, and sterile water were prepared in the lab.

### 2.3 Observation of body weight and fecal water content of mice

At 8:30 am every day (before the experimental operation on the same day, such as before intragastric administration), the weight of the mice was weighed, and fresh fecal samples of each group were collected. One sample of six experimental mice in the same cage in each group was collected, three samples were collected from each group, and the wet weight of the fecal samples was recorded. Samples were then dried to constant weight and recorded as dry weight, and fecal moisture content was calculated. The treatment period was recorded continuously for 7 days.

### 2.4 The serum levels of SP, VIP, and CGRP were detected by ELISA

Whole blood samples (0.7–1.5 mL) of mice in each group were collected using sterilized 2-mL plastic centrifuge tubes. After the blood samples were left for 2 h at room temperature, they were centrifuged at low temperature and high speed (4°C, 3,000 rpm, centrifugation radius 5 cm) for 10 minutes, and the supernatant was obtained. The contents of SP, VIP, and CGRP in serum were determined via ELISA according to the kit instructions.

### 2.5 Extraction of intestinal feces

All mice were sacrificed by cervical dislocation after undergoing gavage treatment. The intestinal feces (from the jejunum to the rectum) of all mice in each group were collected in a sterile environment and stored in a 4°C refrigerator (Li et al., 2024b).

### 2.6 Intestinal microbiota analyzed by 16S rRNA high-throughput sequencing

PCR amplification was realized by Q5 High-Fidelity DNA Polymerase (NEB Company, Ipswich, MA, USA); the extracted DNA was taken as a template to strictly control and minimize the number of amplification cycles but maintain the same amplification conditions. 16S rRNA V4 variable region was used for amplification, and the amplification products were measured by electrophoresis detection; for further fluorescence quantification, the samples were mixed in a corresponding proportion. Using the fluorescent quantitation method of Promega, the recycled products were quantitatively expanded based on the preliminary electrophoretic results; the samples were mixed in a corresponding proportion based on the fluorescent quantitation results. The sequencing library was prepared by Illumina in the following operational approach: first, sequence end repair was performed, the base bulge of DNA sequence 5′ end was removed using End Repair Mix 2 in the kit, and a phosphate group was added to supplement the missing base of 3′ end to ensure that the target sequence was connected to the sequencing joint and fix the DNA molecules on the flow cell. The self-connected fragment was removed, the DNA fragment was amplified by PCR, and the library system after adding the joint was further selected and purified (Li XY. et al., 2022). Sequencing was completed by Beijing Baimaike Biotechnology Co., Ltd. (Beijing, China).

### 2.7 Bioinformatics and statistical analysis

The intestinal microbiota data were processed using the Beijing Baimaike Biotechnology Co., Ltd. cloud platform. The bacterial diversity index (including Chao1, Abundance-based Coverage Estimator (ACE), Simpson, and Shannon) in the intestinal mucosa was measured using MOTHUR (version v.1.30.1, https://www.mothur.org/) based on the operational taxonomic units (OTUs). Principal component analysis (PCA), non-metric multidimensional scaling (NMDS), and linear discriminant analysis (LDA) effect size analysis (LEfSe) were conducted using the R package (https://www.Rproject.org/) to analyze the main distribution characteristics and the similarity of community samples. Functional analysis was conducted to compare the 16S rRNA gene sequence data obtained by sequencing with the Greengenes database. “Mapped” the microbiota composition data to the known gene function profile database to realize the prediction of the metabolic function of the bacterial microbiota.

All data were presented as mean ± standard deviation and analyzed using the SPSS 24.0 software. Independent samples t-test was used to analyze the differences between the two groups. p < 0.05 indicates a significant difference between the two groups, and p < 0.01 indicates a very significant difference between the two groups.

## 3 Results

### 3.1 Changes in general condition, body weight, and fecal water content in mice

**Figure 3:**
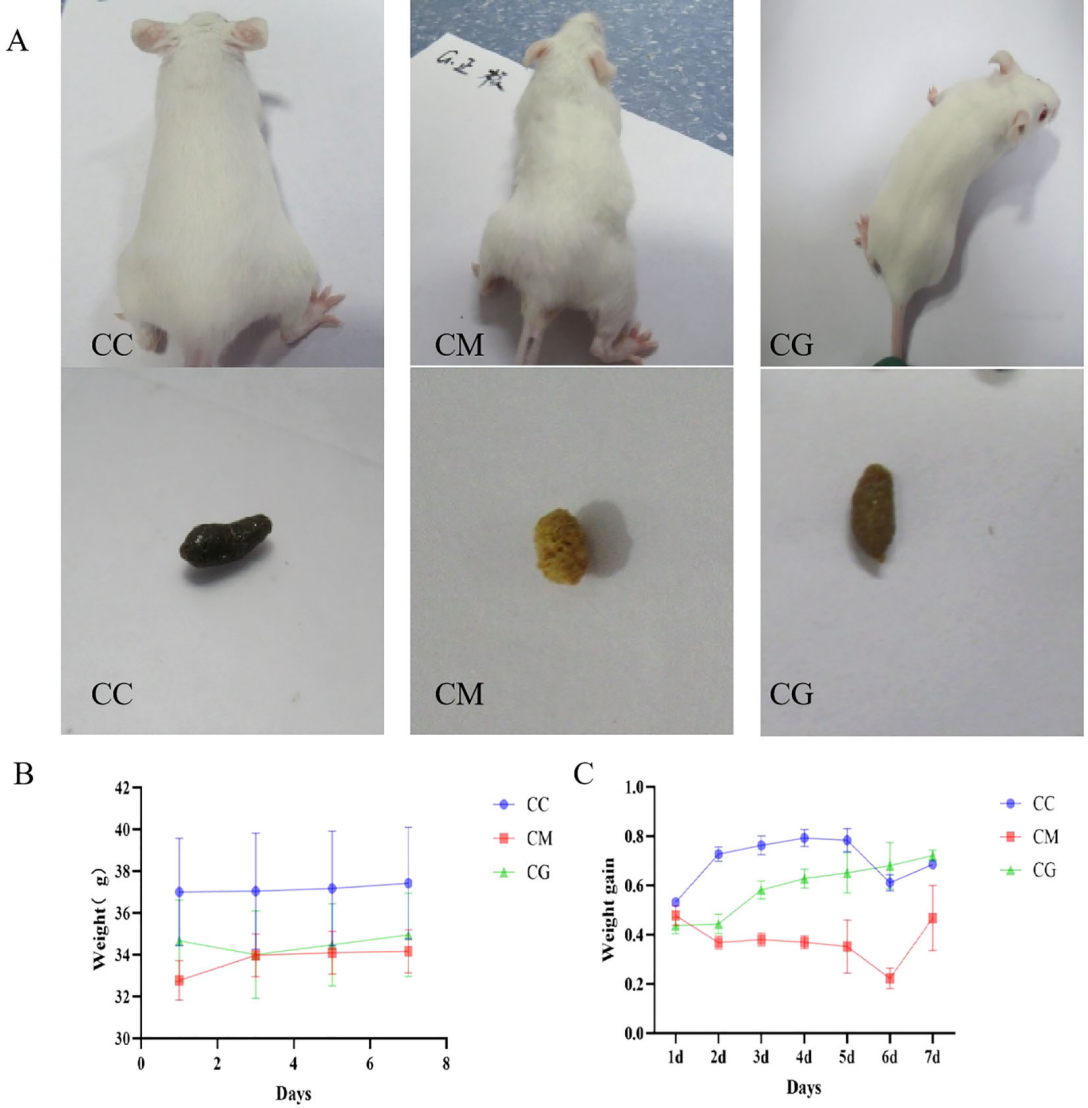
Changes in general condition, body weight, and fecal water content in mice. **(A)** Changes in general condition and fecal morphology of mice. **(B)** Weight changes in mice treated with spleen deficiency constipation of Massa Medicata Fermentata. **(C)** Changes in fecal water content in mice with spleen deficiency constipation treated with Massa Medicata Fermentata.

Before modeling, all three groups of experimental mice exhibited smooth, white, and shiny fur, a sensitive response, pale pink skin, and moderately dry and loose feces forming an oval shape. On the third day, the mice in the model group began to display signs of irritability and nervousness. As the number of modeling days passed, the hair of the mice gradually became messy, withered, and yellow and lost its glossiness. The skin and mucous membranes of the mice also appeared slightly dull and pale. The mice began to exhibit symptoms of spleen deficiency, such as gathering together, arching their backs, appearing tired, decreased mobility, and producing dry and hard feces. After gastric intervention with Massa Medicata Fermentata, the abnormal manifestations of mouse hair, motility, and feces were significantly improved (**Figure 3A**). Mice with spleen deficiency constipation had a significantly lower body weight than those in the control group (p < 0.01). After 7 days of treatment, the weight of the treated groups was not significantly different from that of the control group (**Figure 3B**). As shown in **Figure 3C**, before treatment, the fecal water content of the treated groups was slightly lower than that of the control group. Following treatment, the fecal water content of the CG group was the same as that of the normal group. However, the fecal water content of the CM group decreased even further, which was significantly different from that of both the CC and CM groups (p < 0.01).

### 3.2 Changes in SP, VIP, and CGRP contents in serum

**Figure 4:**
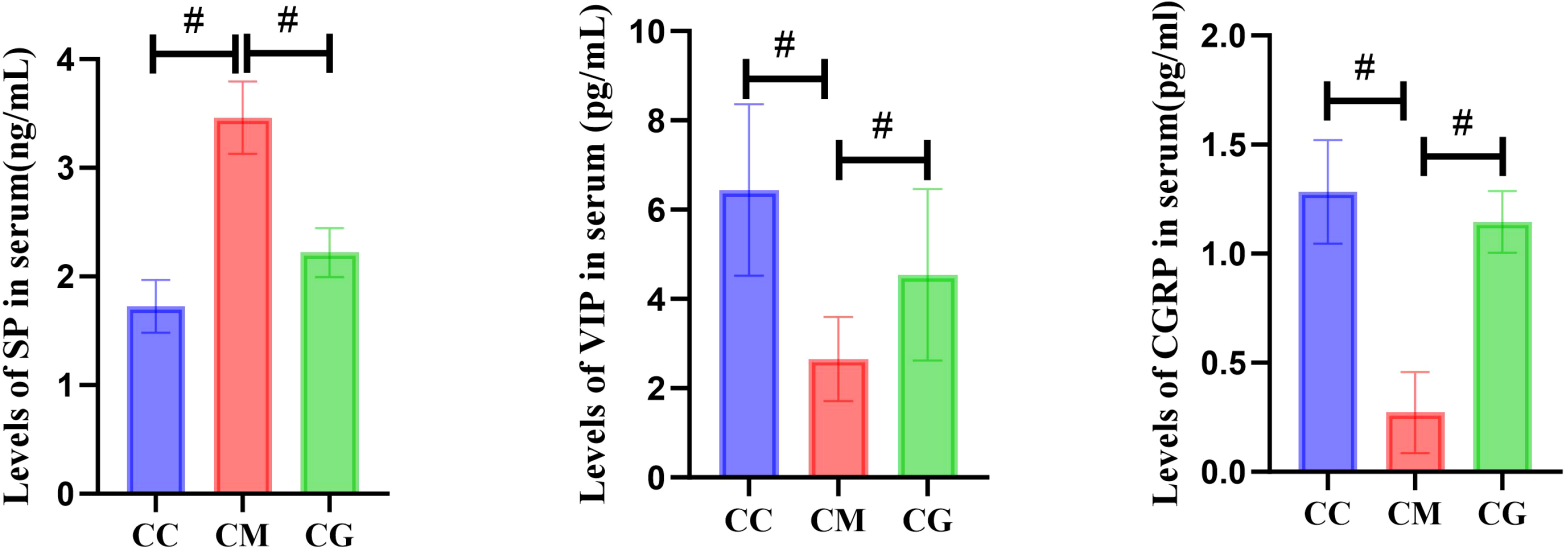
Changes in SP, VIP, and CGRP in serum of mice in each group. SP, substance P; VIP, vasoactive intestinal peptide; CGRP, calcitonin gene-related peptide. "#" represents the comparison between the two groups, p<0.01

Compared with that of the CC group, the content of SP in the serum of the CM group was significantly increased (p < 0.01), while the contents of VIP and CGRP were significantly decreased (p < 0.01). After Massa Medicata Fermentata intervention, the contents of SP, VIP, and CGRP in the serum of spleen deficiency constipation mice with spleen deficiency were all reduced. However, there were still differences in SP content and VIP content between the CC group and CG group (p < 0.05 or p < 0.01) (**Figure 4**).

### 3.3 Quality assessment of intestinal microbiota sequencing data

**Figure 5:**
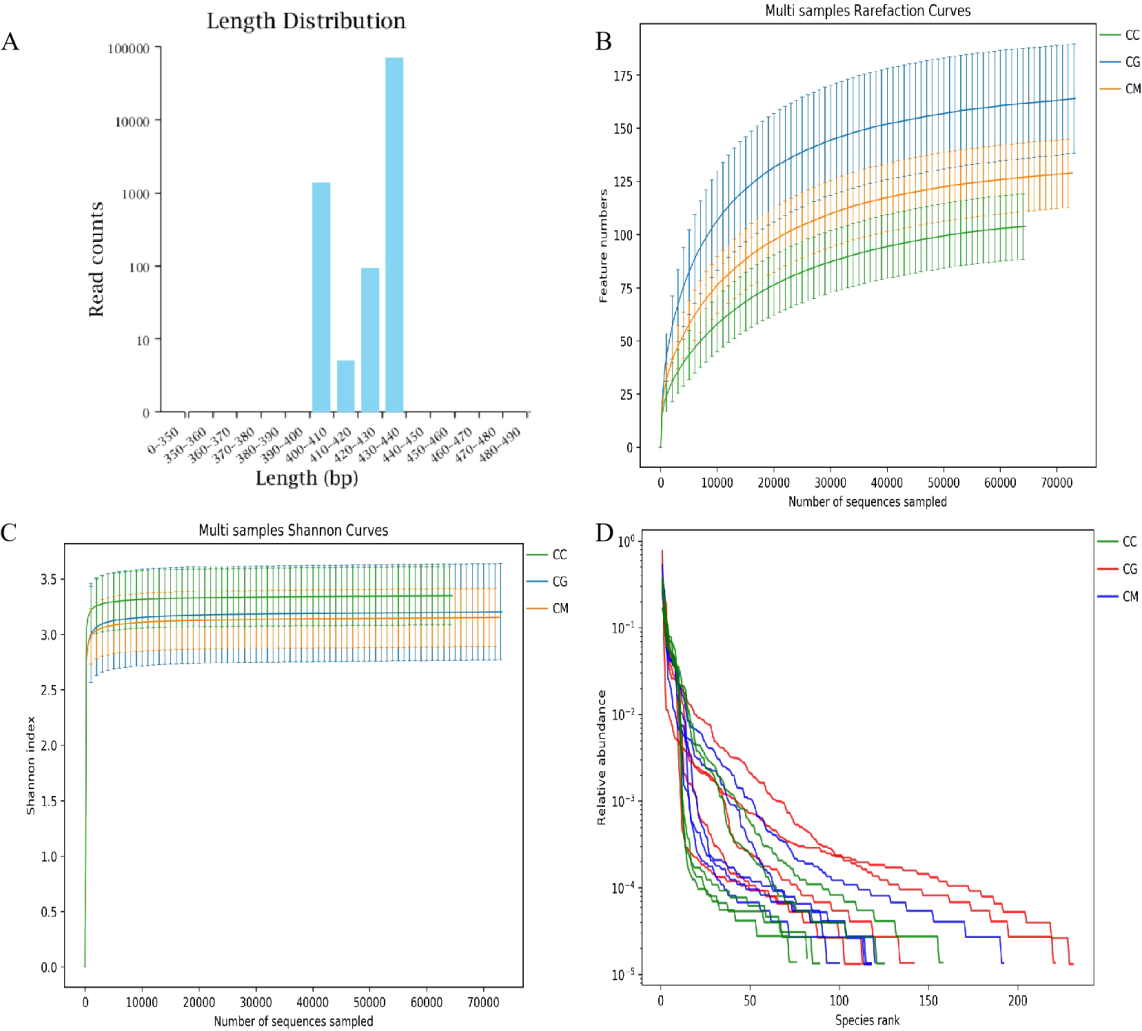
Quality assessment of intestinal microbiota sequencing data. **(A)** Sequence length distribution. **(B)** Chao1 dilution curve. **(C)** Shannon dilution curve. **(D)** Species accumulation curve.

According to the sequence length statistics obtained by this sequencing, the sequence length distribution of each sample was concentrated in the range of 400–440 bp (**Figure 5A**). The dilution curve shows that when the sequencing volume of each sample reached 4,000, the curve entered a plateau, and the microbial quantity detected in each sample was close to saturation (**Figures 5B, C**), indicating that the current sequencing depth was sufficient to reflect the microbial diversity contained in the batch of samples. As shown in **Figure 5D**, the samples continued to increase, the increase rate of species richness slowed down, and the curve exhibited flatness, indicating that species richness no longer increased with the addition of new samples, suggesting that the samples were sufficient to meet the needs of the research. Therefore, we assumed that a reasonable depth of sequencing was applied in this study and that the amount of data sequenced from the samples was sufficient to adequately represent the true appearance of the microbial communities in each sample and could be used for microbial diversity analysis of the batch samples.

### 3.4 OTU number and diversity of intestinal microbiota

**Figure 6:**
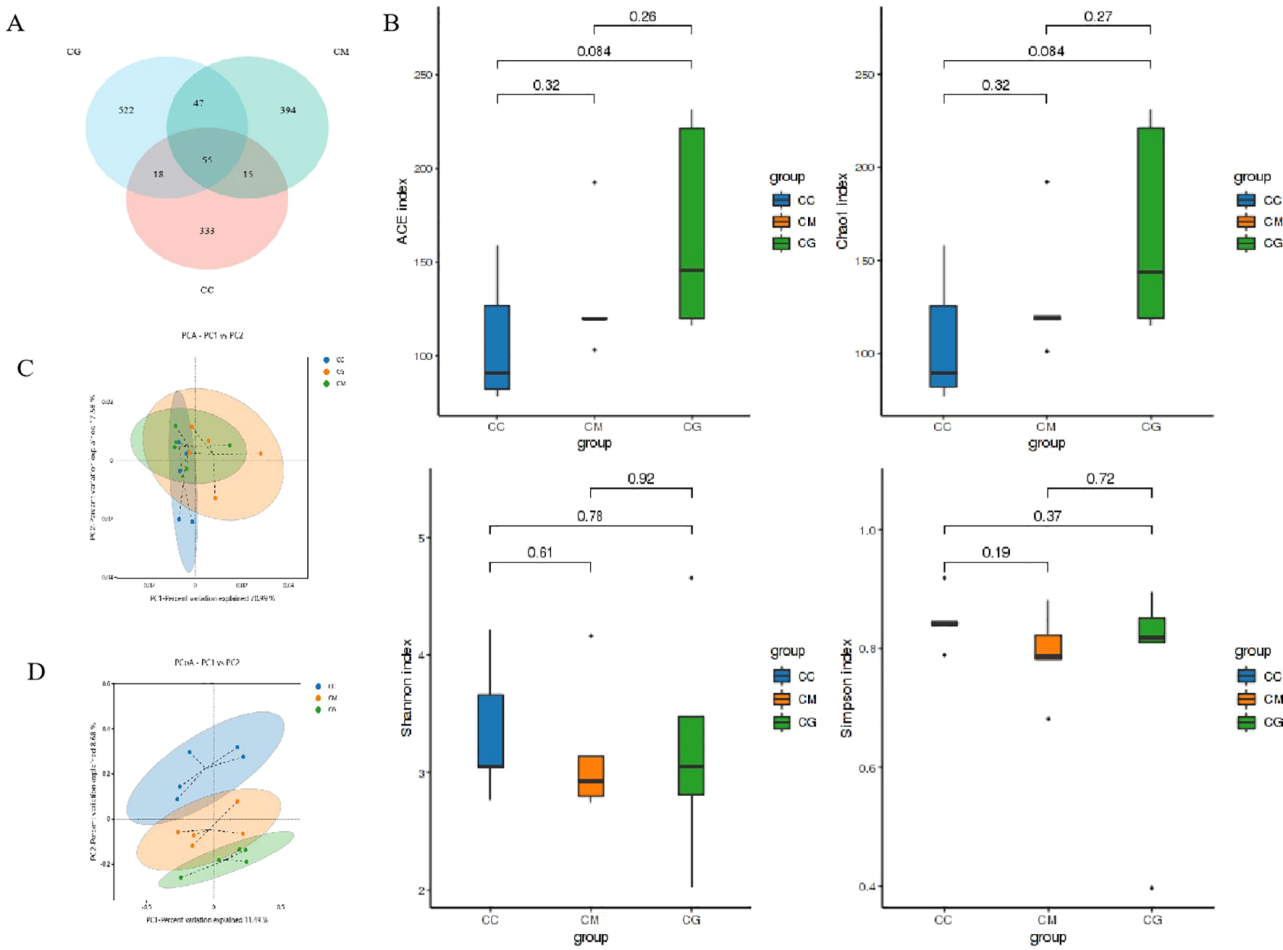
OTU quantity and diversity of microbiota in intestinal contents. **(A)** Venn diagram. **(B)** Alpha diversity index. **(C)** PCA. **(D)** PCoA. OTU, operational taxonomic unit; PCA, principal component analysis; PCoA, principal coordinate analysis.

The total OTUs of the CC, CM, and CG groups were 421, 511, and 642, respectively. The number of OTUs across the three groups was 55, and the total proportion of OTUs among the three groups was 3.49% (**Figure 6A**). To comprehensively evaluate the alpha diversity of microbial communities, ACE and Chao1 indices were used to characterize the richness, and Shannon and Simpson indices were used to characterize the diversity. The larger the index, the higher the total number of communities (Yi et al., 2023b). In the alpha diversity index (**Figure 6B**), ACE, Chao1, and Shannon indices of the CM group were significantly lower than those of the CC group (p < 0.05), indicating that the richness and diversity of intestinal microbiota of mice decreased due to modeling; ACE, Chao1, and Shannon indices of the CG group were all increased after the intervention of Six Shenzhou and even exceeded those of the CC group, indicating that the intervention of Massa Medicata Fermentata regulated the richness of intestinal microbiota to a certain extent and increased the diversity of intestinal microbiota.

Beta diversity describes the differences in species composition between different habitat communities. PCA (**Figure 6C**) and principal coordinate analysis (PCoA) (**Figure 6D**) showed only partial overlap between the CC group and CM group, indicating that constipation changed the microbiota structure of intestinal contents. There was only a small overlap between the CM group and CG group, indicating that Massa Medicata Fermentata affected the microbiota structure of intestinal contents. The close distance between the CC group and CG group suggested that Massa Medicata Fermentata can efficiently restore the microbiota structure of intestinal contents. The above results indicate that the intervention of Massa Medicata Fermentata changes the richness and diversity of the microbiota in the intestinal contents and changes the structure.

### 3.5 The dominant microbiota of intestinal content microbiota

**Figure 7:**
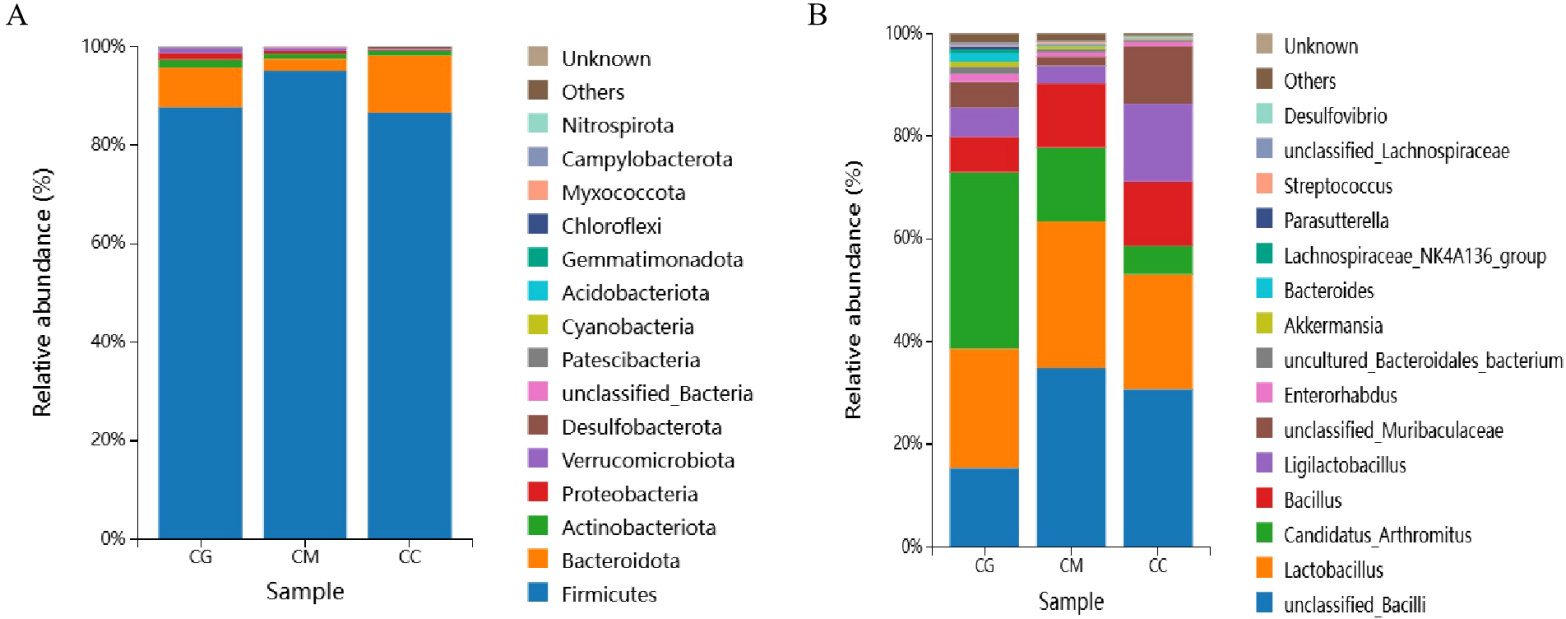
Dominant microbiota of intestinal contents. **(A)** Phylum level. **(B)** Genus level.

The 10 phyla and 15 genera with the highest relative abundance were screened and represented by a bar chart. In the phylum level (**Figure 7A**), Firmicutes is the dominant gate of each group, and the distribution proportion of Firmicutes in the three experimental groups has reached over 80%, followed by Bacteroidota, Actinobacteria, and Verrucomicrobiota in the CM group. Proteobacteria is mainly distributed in the CG group.

At the genus level (**Figure 7B**), *Lactobacillus* is the dominant strain in the CC group, *Bacillus* is the dominant strain in the CG group, and *Candidatus* Arthromitus with metabolic protection is the dominant strain in the CG group. The top eight CC group distributions are *Lactobacillus*, unclassified Bacilli, *Lactobacillus*, *Bacillus*, *Muribaculaceae*, *Candidatus* Arthromitus, *Enterorhabdus*, and *Desulfovibrio*. The top eight CM group distributions are unclassified Bacilli, *Lactobacillus*, *Candidatus* Arthromitus, *Bacillus*, *Ligilactobacillus*, *Muribaculaceae*, *Enterorhabdus*, and *Akkermansia*. The top eight CG group distributions are *Candidatus* Arthromitus, *Lactobacillus*, unclassified Bacilli, *Bacillus*, *Ligilactobacillus*, *Muribaculaceae*, *Bacteroides*, and *Enterorhabdus*.

### 3.6 Characteristic bacteria of intestinal contents

**Figure 8:**
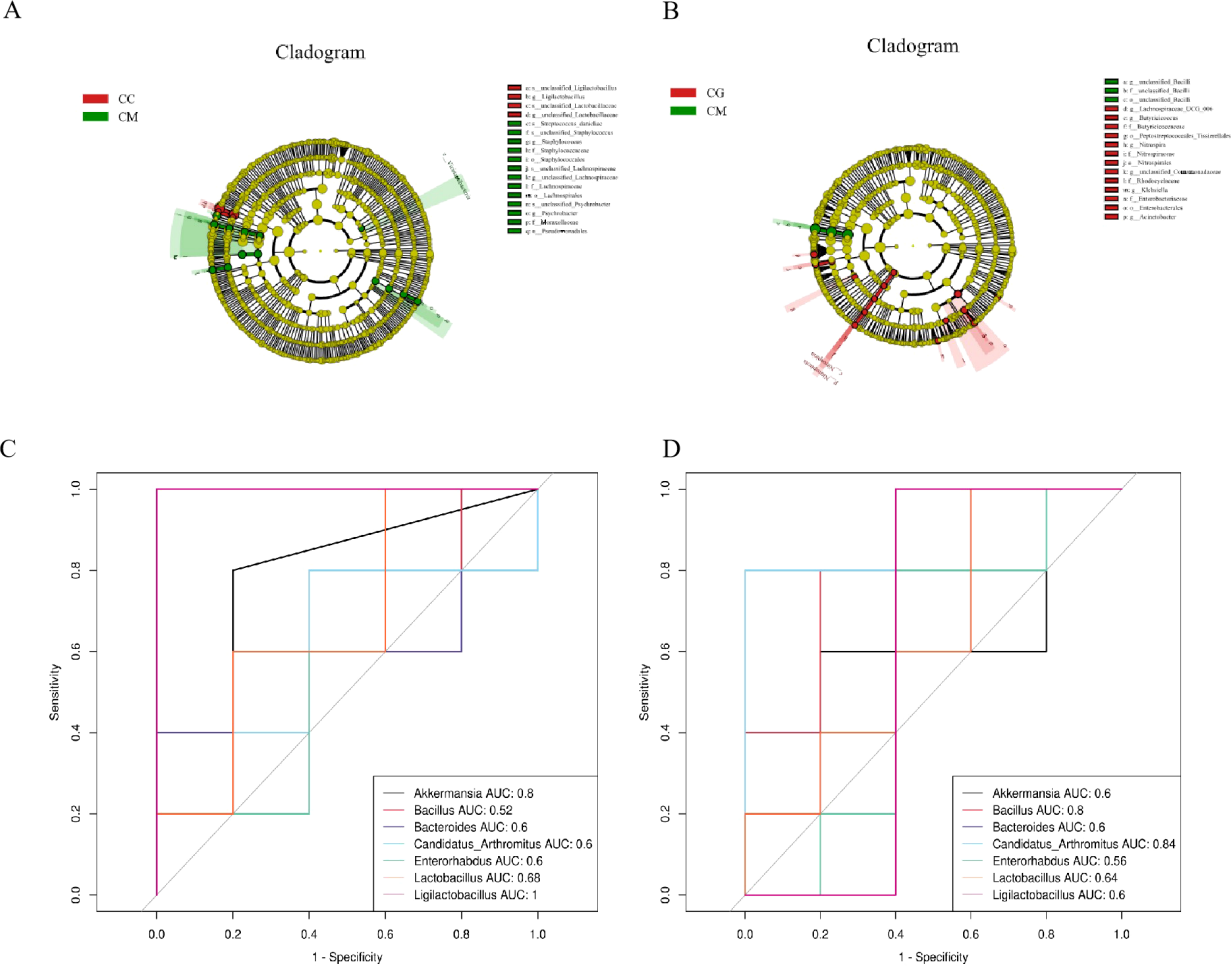
Characteristic bacteria, ROC curve, and correlation analysis of intestinal contents among all groups. **(A)** Characteristic bacteria genera between CC and CM groups. Characteristic bacterial genera between CG and CM groups. **(C)** ROC curve between CC and CM groups. **(D)** ROC curve between CG and CM groups. ROC, receiver operating characteristic; CC, normal group; CM, model group; CG, Massa Medicata Fermentata intervention group.

We selected LEfSe with a logarithmic LDA threshold of 2.0 to identify significantly different microbiota between groups. **Figure 8A** shows the characteristic bacteria between the CC and CM groups, and the characteristic bacteria in the CC group are mainly *Ligilactobacillus* and *Lactobacillus*. **Figure 8B** shows the characteristic bacteria between the CM group and the CG group, and the characteristic bacteria in the CM group is mainly *Bacillus*. The characteristic bacteria enriched in the CG group was *Candidatus* Arthromitus.

The area under the curve (AUC) is the area surrounded by coordinate axes under the receiver operating characteristic (ROC), and AUC is usually 0 to 1. The closer the AUC is to 1, the more likely a microbiota has with relative abundance difference and diagnostic efficacy between the two groups (Yi et al., 2023b). We used 0.7 ≤ AUC < 0.9 as the criterion to verify the accuracy of diagnosis and joint evaluation of characteristic bacteria among different groups and determine whether it has diagnostic efficacy. Characteristic bacteria with AUC ≥ 0.7 were defined as significant bacteria that described different characteristics between the two groups. ROC results showed that *Ligilactobacillus* of the CC group contributed the most at the species level, Bacilli of the CM group contributed the most at the species level, and *Candidatus* Arthromitus of the CG group contributed the most at the species level (**Figures 8C, D**).

### 3.7 Interactions of the characteristic microbiota and environmental factors such as SP, VIP, and CGRP

**Figure 9:**
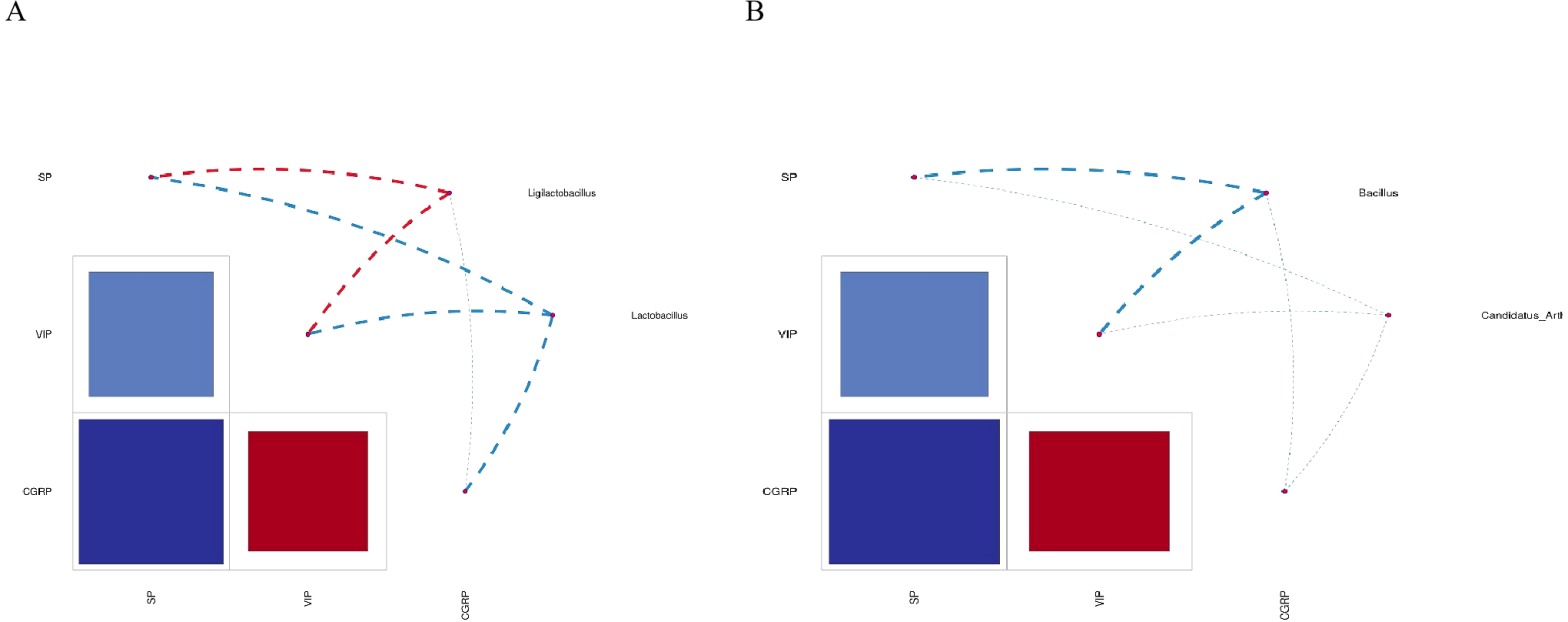
Heatmap of correlation between characteristic bacteria genera and each index in each group. **(A)** Correlation analysis of *Ligilactobacillus*, *Lactobacillus*, SP, VIP, and CGRP. **(B)** Correlation analysis of *Bacillus*, *Candidatus* Arthromitus, SP, VIP, and CGRP. SP, substance P; VIP, vasoactive intestinal peptide; CGRP, calcitonin gene-related peptide.

We performed the correlation analysis of SP, VIP, and CGRP in the characteristic microbiota and serum and drew a heatmap of the correlation network, as shown in **Figure 9**, *Ligilactobacillus* was positively correlated with SP, positively correlated with VIP, and negatively correlated with CGRP. *Lactobacillus* was negatively correlated with SP, VIP, and CGRP. *Bacillus* was negatively correlated with SP, VIP, and CGRP. *Candidatus* Arthromitus was negatively correlated with SP, VIP, and CGRP.

## 4 Discussion

There are many microorganisms in the human gut, which reside in the human gastrointestinal tract and interact with the host, playing an important role in the health of the host (Ali et al., 2021), including changing or affecting the host’s metabolic phenotype and immune system, maintaining host energy homeostasis, and preventing disease susceptibility. As research into the microbiome advances, the notion of a “microbiome-free” paradigm within neuroscience has undergone progressive revision, notably with the emergence of the brain–gut axis concept. This axis represents a bidirectional information exchange system that integrates the functions of the brain and intestine. Consequently, the role of microbiota in nervous and mental disorders has garnered increasing attention from researchers (Wang et al., 2019). This concept was first proposed in the 1980s when Jeong et al. Studied the regulatory effect of bombesin on cholecystokinin (Jeong et al., 2019). There is a close relationship between the central nervous system (CNS) and intestinal microbiota, and the normal function of the CNS is of great significance to the microenvironmental homeostasis of intestinal microbiota. Two aspects are involved: on the one hand, regulating the composition of the enteric nervous system (ENS) or intestinal microbiota and the abundance of related microbiota can play a role in brain development and function; on the other hand, the brain also regulates gut function and the presence of intestinal microbiota, and each component of this complex network regulates and manipulates the other systems involved. The brain–gut axis connects the brain to the gut in three main ways, namely, the neuroendocrine pathway, immune pathway, and metabolic pathway, involving the gastrointestinal tract, autonomic nervous system (ANS), ENS, CNS, vagus nerve, endocrine system, and immune system (Guo et al., 2020). Brain–gut peptide is an essential molecular basis of the brain–gut axis. It and its related signal molecules control the changes in gastrointestinal motility through a bidirectional regulatory pathway between the CNS and ENS. Intestinal microbiota disturbance, diversity reduction, and brain–gut axis disturbance are potential pathogenesis of spleen deficiency constipation. At present, spleen deficiency constipation has been widely studied in the academic world, almost from the level of the brain–gut axis, including the involvement of the hypothalamic–pituitary–adrenal (HPA) axis and autonomic nervous system, activation of the immune response, comorbidity with anxiety and depression, increased interoception, and changes in neuralgia processing (Yu, 2020; Li et al., 2024a). In this study, it was found that compared with the CC group, the intestinal microbiota structure of the CM group showed a significant difference, and the microbial diversity was significantly reduced. The body’s immune response is activated, and it is in a low-grade inflammation state for a long time. Mice in the CM group showed strong susceptibility to mental illness and stress, which was also an important co-factor promoting spleen deficiency constipation. All these indicate that spleen deficiency constipation is a disorder at various levels of the brain–gut axis, reflecting that the onset of spleen deficiency constipation is closely related to the brain–gut axis.

The results of this study showed that the level of cerebral intestinal peptide SP decreased significantly and that the levels of VIP and CGRP increased significantly in mice with spleen deficiency constipation. After the intervention of Massa Medicata Fermentata, SP, VIP, and CGRP all decreased somewhat. SP is a major excitatory neurotransmitter in the gastrointestinal tract. It plays a role in the regulation of gastrointestinal motor function mainly through the activation of the spirocyte pathway (Elsalhy et al., 2013). It is also the first brain–intestinal peptide with multiple distributions confirmed, consisting of 11 amino acids, and distributed in the CNS, the dorsal root of the spinal cord, and ENS (Layunta et al., 2018; Fukudo et al., 2021). Previous studies have shown that compared with the normal population, plasma SP content in functional constipation patients is significantly reduced, and SP content is significantly increased after drug intervention, which can effectively improve the gastrointestinal sensory threshold and eliminate gastrointestinal allergies (Hu et al., 2020; Qiao et al., 2024). The results of this study suggest that the serum SP level of mice with spleen deficiency constipation decreased; after the intervention of Massa Medicata Fermentata, the SP level increased; the purpose of treating functional habit constipation of spleen deficiency type was achieved by regulating gastrointestinal motor function. VIP is a neuropeptide that inhibits gastrointestinal movement mainly by stimulating the production of nitric oxide in gastrointestinal smooth muscle cells and increasing the concentration of cyclic adenosine and cyclic guanosine phosphate in cells (Lu, 2020). It has dual functions of neurotransmitter and neuromodulation, mainly including it is expressed in the central nervous system and gastrointestinal tract. Various gastrointestinal diseases include irritable bowel syndrome and reflux esophagitis (Rattan, 2005; Black and Ford, 2018). Dyspepsia is associated with abnormal VIP. Studies have shown that the regulation of the ENS in healthy people may be related to the nerve relaxation response of VIP (Iwasaki et al., 2019). Plasma VIP level can be significantly increased in constipation patients, and gastric motility such as disturbance of gastric electric rhythm and slowing of gastric emptying rate can be significantly weakened (Sun et al., 2019; Guo et al., 2024). In this study, the serum VIP level of mice with spleen deficiency constipation was significantly higher than that of the normal group, and the VIP level was significantly reduced after the intervention of Lishenqu. The results indicated that VIP was closely related to the occurrence of spleen deficiency constipation; meanwhile, Massa Medicata Fermentata could improve gastric motility by changing the VIP level of serum midbrain intestinal peptide. CGRP mainly has two subtypes, α-CGRP and β-CGRP, which are widely distributed in capsaicin-sensitive primary afferent fibers and are distributed in both the gastrointestinal tract and the central nervous system (Ulusoy et al., 2021). It is not only a gastrointestinal hormone but also a neuropeptide that plays an important regulatory role in the brain–gut axis mechanism (Liu et al., 2019; Wang et al., 2023). It mainly plays an inhibitory role in the gastrointestinal tract, which is manifested by reducing the secretion of gastric acid and inhibiting gastrointestinal inflammation (Li et al., 2021). In this experiment, by assessing the serum CGRP levels of mice in each experimental group, it was observed that the CGRP level significantly increased in the CM group and notably decreased post-treatment. These findings corroborated earlier results, suggesting a close association between CGRP and the onset of spleen deficiency constipation. Moreover, it indicated that Massa Medicata Fermentata could potentially alleviate spleen deficiency constipation by reducing serum CGRP levels.

The human gut is a huge and complex microecosystem (Zhao et al., 2023; Zhou et al., 2024). Bacteria interact with each other to participate in the growth and development process of the organism and maintain the balance of intestinal microecology and the stability of the organism (Wang et al., 2020). The occurrence and development of many gastrointestinal diseases are also directly or indirectly related to changes in the microbiota (Guo et al., 2005). The disturbance and decrease of intestinal microbiota diversity have been considered one of the potential pathogenesis of spleen deficiency constipation (Liu et al., 2024; Wu et al., 2024). We analyzed the intestinal microbiota of experimental mice in each group, and the results showed that the intestinal microbiota diversity of mice with spleen deficiency constipation decreased and that the dominant microbiota and characteristic microbiota changed, suggesting that the intestinal microbiota was unbalanced. After the intervention of Massa Medicata Fermentata, the intestinal microbiota diversity of spleen deficiency constipation mice increased; the dominant microbiota became *Candidatus* Arthromitus, *Lactobacillus*, unclassified Bacilli, *Bacillus*, *Ligilactobacillus*, *Muribaculaceae*, *Bacteroides*, and *Enterorhabdus*; and the characteristic microbiota became *Candidatus* Arthromitus. We found that *Ligilactobacillus* was positively correlated with SP and that *Ligilactobacillus* was negatively correlated with VIP. *Candidatus* Arthromitus was negatively correlated with CGRP. This was consistent with the results of SP, VIP, and CGRP levels measured in serum.

*Ligilactobacillus* secretes antimicrobial molecules, such as organic acids, ethanol, and reuterin. Due to its antibacterial activity, *Ligilactobacillus* inhibits the customization of pathogenic microorganisms and perhaps the composition of the symbiotic microbiome in the host (Li et al., 2023; Liu et al., 2023; Fang et al., 2025). *Ligilactobacillus* and *Candidatus* Arthromitus both increase the content of butyrate in the gut, and butyrate plays a broad role in local and whole-organism signaling networks by binding to G protein-coupled receptors (GPCRs) (Hurst et al., 2014; Li CR. et al., 2022). These effects include enhanced intestinal barrier function, mucosal immunity, and intestinal homeostasis (Ge et al., 2017). In turn, these effects can improve energy metabolism, promote weight loss, reduce inflammation, and allow the gut–brain axis to function properly (Rindeau et al., 2003; Soret et al., 2010).

In summary, intestinal microbiota imbalance with spleen deficiency constipation is mainly manifested as a decrease in the number of probiotics, an increase in the number of harmful bacteria, a sharp decline in serum SP level, and an increase in VIP and CGRP levels. By regulating the structure of intestinal microbiota, Massa Medicata Fermentata affects the secretion of short-chain fatty acids in the intestine and regulates intestinal function, while short-chain fatty acids stimulate the secretion of serum peptides, making the brain–intestinal axis operate normally, forming a virtuous cycle, and thus alleviating constipation.

## Data Availability

The data presented in the study are deposited in the NCBI repository, accession number is PRJNA1226784.
